# Outcomes of co-designed communities of practice that support members to address public health issues

**DOI:** 10.1093/heapro/daae080

**Published:** 2024-07-11

**Authors:** Sanne H Elbrink, Shandell L Elmer, Melanie H Hawkins, Richard H Osborne

**Affiliations:** School of Health Science, Centre for Global Health and Equity, Swinburne University of Technology, John Street, Hawthorn, Victoria 3122, Australia; Durham University Business School, Durham University, Mill Hill Lane, Durham DH1 3LB, UK; School of Health Science, Centre for Global Health and Equity, Swinburne University of Technology, John Street, Hawthorn, Victoria 3122, Australia; School of Nursing, College of Health and Medicine, University of Tasmania, Locked bag 1322 Newham, Launceston, Tasmania 7250, Australia; School of Health Science, Centre for Global Health and Equity, Swinburne University of Technology, John Street, Hawthorn, Victoria 3122, Australia; School of Health Science, Centre for Global Health and Equity, Swinburne University of Technology, John Street, Hawthorn, Victoria 3122, Australia

**Keywords:** communities of practice, knowledge sharing, knowledge translation, public health co-design

## Abstract

Communities of practice are commonly used to support members in responding to public health issues. This study evaluated the outcomes of five co-designed communities of practice to determine if members’ expectations were met, if knowledge sharing between members extended to knowledge translation, and if that supported members in addressing public health issues. Data were collected through an initial needs assessment, observations were made during community of practice sessions over 1 year, and qualitative interviews were conducted at the end of that year. The findings provided evidence that members’ expectations were met, knowledge sharing took place within the communities of practice, and personal benefits gained supported members in advancing knowledge sharing with other members to knowledge translation outside their community of practice. Results demonstrate three outcomes of knowledge translation for members: disseminating knowledge to others, applying knowledge to make small-scale changes in practice and leveraging the knowledge to expand its reach beyond members’ organizations. While the scale and speed of expanding outcomes were below initial expectations as indicated in the initial needs assessments, members remained optimistic about achieving larger-scale impacts in the future. This study showed that communities of practice achieve gradual progress rather than quick wins. Co-design supports the facilitators in meeting members’ needs, which can positively contribute to members sharing knowledge and translating that knowledge to support their practice to address public health issues.

**Contribution to Health Promotion**

- This study shows that knowledge shared *within* and subsequently translated *outside* communities of practice supports members in addressing public health issues.
- Community of practice members: (1) disseminate shared knowledge to others, (2) apply knowledge to change practice and, sometimes, (3) leverage knowledge to expand its reach beyond their organization.
- Co-designing communities of practice may support policymakers, professionals and researchers to establish communities of practice that meet members’ needs, go beyond knowledge sharing to facilitate knowledge translation and enable outcomes that support members’ responses to public health issues.

## BACKGROUND

To address public health issues and achieve better health outcomes, we require effective methods that take account of both micro- and macro-level determinants of health (Paskett *et al.*, 2016; World Health Organization, 2022). Communities of practice—groups who share and deepen their knowledge through interactions (Wenger *et al.*, 2002)—hold the potential to facilitate knowledge translation processes across organizational boundaries and effectively address public health issues (Kothari *et al.*, 2011; Jennings Mabery *et al.*, 2013). Previous studies have identified possible knowledge translation outcomes for communities of practice, including members disseminating knowledge to their parent organization, using knowledge for practice change and improving health outcomes (e.g. Chandler and Fry, 2009; Jennings Mabery *et al.*, 2013; Poole and Bopp, 2015). Robust evaluations are needed to confirm these outcomes, including systematic descriptions of knowledge translation across and beyond communities of practice focusing on public health issues (Barbour *et al.*, 2018; James-McAlpine *et al.*, 2023).

Communities of practice typically focus on knowledge sharing and learning (Wenger *et al.,*2002). However, for effective responses to public health issues, members must process knowledge shared *within* communities of practice and translate that knowledge for practical utilization *outside* communities of practice. This complex and multifactorial process can be described by utilizing a Knowledge-to-Action (KTA) framework (Graham *et al.*, 2006; Field *et al.*, 2014; Rimmer *et al.*, 2016). Knowledge sharing *within* communities of practice involves members obtaining and passing on knowledge. The obtained knowledge is then processed to create new, tailored knowledge (Nonaka *et al.*, 1996; Collins, 2010). Explicit knowledge is shared through presentations, lectures, documents and videos, while members share tacit knowledge through informal interactions, such as storytelling, sharing experiences, best practices or brainstorming. Both explicit and tacit knowledge are seen as potentially important for knowledge sharing in communities of practice (Polanyi, 1966; Kothari *et al.*, 2011; Jennings Mabery *et al.*, 2013). Knowledge translation usually occurs *outside* communities of practice, where members synthesize, appraise, adapt and tailor the knowledge to their specific contexts, such as their parent organizations (Gabbay *et al.*, 2003; Roscoe, 2004; Graham *et al.*, 2006; Rimmer *et al.*, 2016).

In communities of practice that evolve spontaneously among like-minded people, knowledge translation outcomes have been identified, particularly in those communities related to urgent public health issues. These spontaneous groups often tend to naturally fulfil members’ existing needs (e.g. Mullan *et al.*, 2022; Sant Fruchtman *et al.*, 2022). To fulfil members’ needs in top–down initiated communities of practice, facilitators can approximate these spontaneously evolved groups by deliberately drawing together people with shared interests and implementing actions to ensure members’ needs are met (Field *et al.*, 2014). Co-design—the joint design of communities of practice with members, initiators and facilitators—has been suggested as a way to achieve this (Bindels *et al.*, 2014; Dijkmans-Hadley *et al.*, 2015). However, co-design of communities of practice is uncommon, with top–down initiated and run communities of practice being the most common practice (Elbrink *et al.*, 2021).

Our previous work developed a method with a needs assessment for prospective members to co-design their community of practice to meet members’ needs related to addressing specific public health issues such as (mental) health literacy and trauma-informed care (Elbrink *et al.*, 2023). The current study evaluated the application of this novel co-design method through observation and interviews in four communities of practice. We sought to (i) identify outcomes that address specific public health issues among members who participated for over 1 year, and (ii) examine if the novel co-design method fulfilled members’ needs. We expected that with an understanding of tangible outcomes, the initiators and facilitators will be more confident with the expected impact of their efforts, and managers may choose to support their employees to participate in communities of practice (Wenger *et al.*, 2002; Elbrink *et al.*, 2023).

## METHODS

**Fig. 1:**
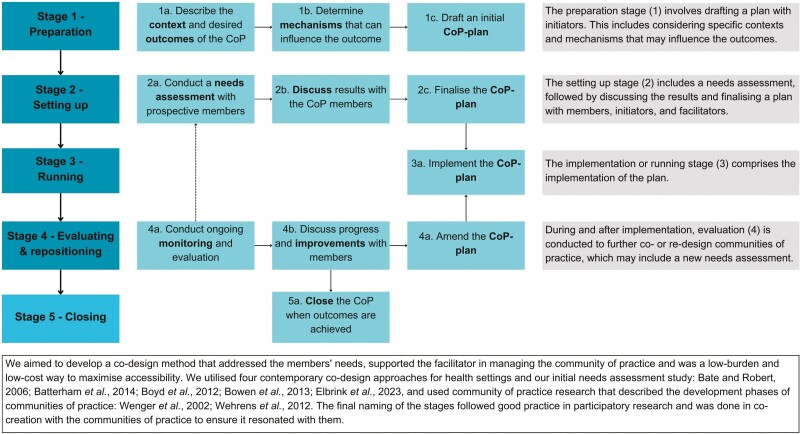
Summary of co-design method for communities of practice

This study used qualitative methods to determine the outcomes experienced by members of co-designed communities of practice focused on public health issues such as (mental) health literacy or trauma-informed care. The evaluation included interviews and observations that compared the observed outcomes with participants’ expectations recorded in a needs assessment applied at the start of the study (Elbrink *et al.,* 2023). Previous studies suggested co-design as a possibility for designing communities of practice that address members’ needs (Bindels *et al.*, 2014; Dijkmans-Hadley *et al.*, 2015). Some studies involved minor co-design elements and were typically time-consuming for members and facilitators (Mazer *et al.*, 2015; Toledo-Chávarri *et al.*, 2020; Livergant *et al.*, 2021). The development of the co-design method was not a part of this study yet is summarized here for reference (Figure 1).

### Sampling study settings and research participants

This study examined five communities of practice (referred to as CoP A, B, C, D and E). These communities of practice were all initiatives of external programmes, separate from the focus of this study. R.H.O. initiated CoP A and D to support existing Australian and international initiatives. CoP B was an intervention within a major project involving R.H.O. and S.L.E., but neither was involved in CoP B. The initiator of CoP C was a professional associate of S.L.E. The initiator of CoP E was a temporary member at the start of CoP B. All the CoP initiators proactively approached the lead researcher (S.H.E.) to join this study. Communities of practice were only included in the study if they addressed a specific public health issue and involved members from organizations who joined voluntarily (Elbrink *et al*., 2023).

Due to geographical barriers and regulations related to COVID-19, the study was restricted to online communities of practice. The number of communities of practice in this study was limited to five to ensure a balance between having a diversity of settings and a manageable amount of data. Recruitment ceased once five communities of practice were implemented. The communities of practice shared similarities and differences; for example, they all met in online sessions but differed in which platforms they used. Table 1 provides an overview of the similarities and differences.

**Table 1:** Characteristics and differences of the five communities of practice

Characteristic	Similarities	Differences
1. Members	Voluntary joining of people Largely consisting of professionals Members are not commonly known to each other Open to new members	CoP C and E included members who self-identified as having lived experience.
2. Establishment	Top–down initiative to co-design the community of practice with the members	Members of CoP A and B are directly related to the implementation of projects. However, team members are part of individual project teams. CoP E is part of the implementation plan of a project. CoP C and D are independent of other projects.
3. Structure	Not intraorganizational (only members of one organization) Multidisciplinary Structured facilitation	CoP A and B are interorganizational CoP C, D and E have independent members from different organizations CoP C and E have rotating facilitation CoP A is a global CoP^a^ CoP B, C and E are state-wide CoPs CoP D is a national CoP
4. Interaction	Online No notable interaction outside of meetings	Specific interaction choices were dependent on outcomes needs assessment
5. Public health issue	Focus on action on one or more public health issues The aim of initiating organization is communicated with the CoP	Topic CoP A: Health literacy and NCDs Topic CoP B: Mental health literacy Topic CoP C and D: Health literacy Topic CoP E: Mental health (Trauma Informed Care)
6. Role of the research team in the CoPs.	Involvement of the lead researcher (SHE) in the preparation, including needs assessment, stage of the communities of practice through discussions with the facilitator(s) Occasional brainstorm discussions with facilitators during the implementation stage of the communities of practice	CoP A and D were facilitated by RHO. SHE provided administrative support (e.g. setting up meeting, distributing recordings). MH and SLE occasionally joined sessions of CoP A and D as being interested in the topic of the CoP. SLE joined CoP B and C once as a topic expert.

^a^Participating countries: Australia, Benin, Brunei, Cameroon, Canada, Denmark, Egypt, England, France, India, Ireland, Mali, Netherlands, Norway, Portugal, Scotland, Slovakia and Spain.

All potential members were informed they could voluntarily opt-in to be observed. Demographic data were not obtained from potential members to minimize the imposition on members. Observations of the five communities of practice started at the first session and continued, where possible, for 1 year to collect data on potential knowledge translation outcomes (CoP A, B, C and D). The coordination of CoP E was handed over to an independent planning committee nine months into the study, which led to the cessation of observations. Diverse interviewees were purposefully selected to ensure a balanced representation, considering aspects such as facilitators and members, participation in the needs assessment, observed active participation levels and expertise on the topic (Table 2). Interviews were conducted until saturation was reached and no further outcomes emerged (Carminati, 2018).

**Table 2:** Overview of the communities of practice and the participant selection

	Sessions observed	Unique CoP members	Average members per session	Study period	Number of interviews	Interviewee members—facilitators	Number of non-responses—declines	Interviewee needs assessment yes—no	Interviewee active—passive	Interviewee Expert—non expert
CoP A	8	51	20	Nov 21—Feb 23	6	5—1	1—0	4—2	4—2	3—3
CoP B	12	36	10	Feb 21—Feb 22	6	5—1	0	4—2	3—3	2—4
CoP C	6	41	16	Sep 21—Aug 22	7	4—3	3—0	4—3	4—3	3—4
CoP D	10	96	24	Sep 21—Aug 22	6 (7)^a^	6—0	2—2	5—1	3—3	2—4
CoP E	9	154	43	Nov 21—Jul 22	7	5—2	2—1	4—3	4—3	4—3
Total	45	378			32	25—7	8—3	21—11	18—14	14—18

^a^The facilitator of CoP A was the same as CoP D and is only included once in the statistics under CoP A.

### Data collection

Data collection aligned with the timeline of each community of practice. Observations commenced with the first session, during which the needs assessment results were presented. Members discussed their collective needs, aims, expectations and preferred ways of interacting and agreed on the ways of working. The sessions of CoP A, B, C and D were recorded and transcribed. The lead researcher (S.H.E.) attended the sessions as an observing participant and took notes. Due to the specific setting of CoP E (trauma-informed care), sessions were not recorded, and only notes were taken.

Interviews with members and facilitators were conducted in the 2 months following the end of observations. A semi-structured topic list was used to explore outcomes within and outside the communities of practice. Additionally, members were asked about the impact of these outcomes on their practice and whether they observed variations across different settings.

### Data analysis

The interview data were thematically analysed using standard qualitative approaches and support of Nvivo12 (Braun and Clarke, 2012). Quotes were identified and classified as outcomes occurring within or outside of communities of practice. The quotes were deductively coded against outcomes abstracted and synthesized from previous studies: (i) knowledge sharing within communities of practice, (ii) disseminating knowledge to others in the parent organization, (iii) using knowledge in practice and (iv) improving health outcomes through systemic changes (e.g., Chandler and Fry, 2009; Jennings Mabery *et al.*, 2013; Poole and Bopp, 2015). This synthesis is reported in a realist synthesis currently undergoing review elsewhere and can be obtained upon request from the corresponding author. After the deductive coding, we explored if and how the outcomes mentioned by the interviewees revised or extended the identified outcomes from previous studies. Interviewees’ quotes were hierarchically organized into main outcomes, which consisted of multiple sub-outcomes. All outcomes were clearly defined and reviewed again by the research team to ensure the quotes from the interviews consistently represented the outcome. Finally, we reviewed the quotes again to determine on which level—micro (individual), meso (organizational) or macro (societal)—the outcome created an impact.

Transcripts and notes from the observations were deductively analysed to code them to the identified outcomes. This was done by searching for the identified outcomes, including synonyms, in field notes and transcripts with the support of Nvivo12. Quotes from the field notes and transcripts were recorded if they complemented outcomes, and notes were taken if no support or objections were found for specific outcomes.

The last part of the analysis examined if and how observed outcomes coincided with members’ initial expectations. The needs assessments previously identified the following short- and long-term outcomes for members of communities of practice: developing increased knowledge about the topic, taking action to change practice, building or having connections, improving health outcomes and receiving support from other members (Elbrink *et al.*, 2023).

### Research team involvement and reflexivity

The research team was in some way involved in all communities of practice, which had the potential to influence study outcomes. Since we could not objectively assess the influence, the potential influence was self-assessed. The influence from the lead researcher (S.H.E.) at the start of the sessions was assessed as high for CoP A, D and E, medium for CoP C and low for CoP B. The influence of S.H.E. during the sessions, after the start, was self-assessed as high for CoP A and D, medium for CoP B and low for CoP C and E. The influence stemmed from conversations between SHE and facilitators outside the sessions. The lead researcher observed all sessions without active participation, providing information about this study only during the sessions. R.H.O. facilitated CoP A and CoP D and was one of the interviewees. S.H.E. conducted all interviews and debriefed with S.L.E. After all data collection was concluded, S.H.E. undertook the initial data analysis and discussed results and interpretations with the research team.

### Ethical statement

Ethical approval for this study was obtained from the Human Research Ethics Committee of Swinburne University of Technology. Study participants provided written consent prior to the community of practice commencement. Interviewees provided their written consent before the interviews. Data were deidentified by assigning each participant a unique participant identification number.

## RESULTS

A total of 6–12 sessions of each community of practice (a total of 45 sessions) were observed for a year, involving 378 unique members, of which 246 consented to be observed as part of this study. The community of practice sessions had an average attendance of 25–40% of the total number of signed-up members. The first session of each community of practice had a much higher number of members than subsequent sessions. Over time, the attendance rate per session stabilized to a core group that participated regularly and others that engaged occasionally (peripheral members). From 10% to 15% of the signed-up members attended just one session. CoP B and C had relatively large core groups and few peripheral members. CoP A, D and E were the opposite, with a small core group and a large group of peripheral members. Forty-three members, facilitators and initiators were invited for the evaluation interviews. Thirty-two agreed to be interviewed, four declined and seven did not respond after one reminder email. Table 1 provides an overview of the characteristics of the communities of practice.

The qualitative analysis of interviews identified 264 outcomes of knowledge sharing and translation, which were categorized into five main outcomes (Outcomes a, b, c, d and e—see below). Guided by the KTA framework, the outcomes were divided into those occurring *within* and *outside* communities of practice. Two micro-level outcomes of knowledge sharing occurred *within* communities of practice through activities and participation. Members (a) transferred (passed on) knowledge to other members and, when they obtained knowledge, they created individual, customized knowledge and increased their capacity. Participation in communities of practice also (b) generated personal benefits in individual members. These personal benefits supported direct engagement in knowledge sharing and, indirectly, the undertaking of knowledge translation.

The knowledge-sharing outcomes directly supported three knowledge translation outcomes *outside* communities of practice. The first outcome occurred on the micro-meso level, where (c) members disseminated knowledge shared within communities of practice to others through interactions outside communities of practice. This dissemination to others in and outside parent organizations influenced the second meso-level knowledge translation outcome, (d) where members applied knowledge and made changes in their practice. This second outcome sometimes influenced a third macro-level outcome, (e) where members leveraged the knowledge to expand its reach beyond their parent organization. The loop was closed when members brought their experiences from *outside* the community of practice back to the sessions, thereby enhancing knowledge sharing *within* communities of practice.

**Fig. 2:**
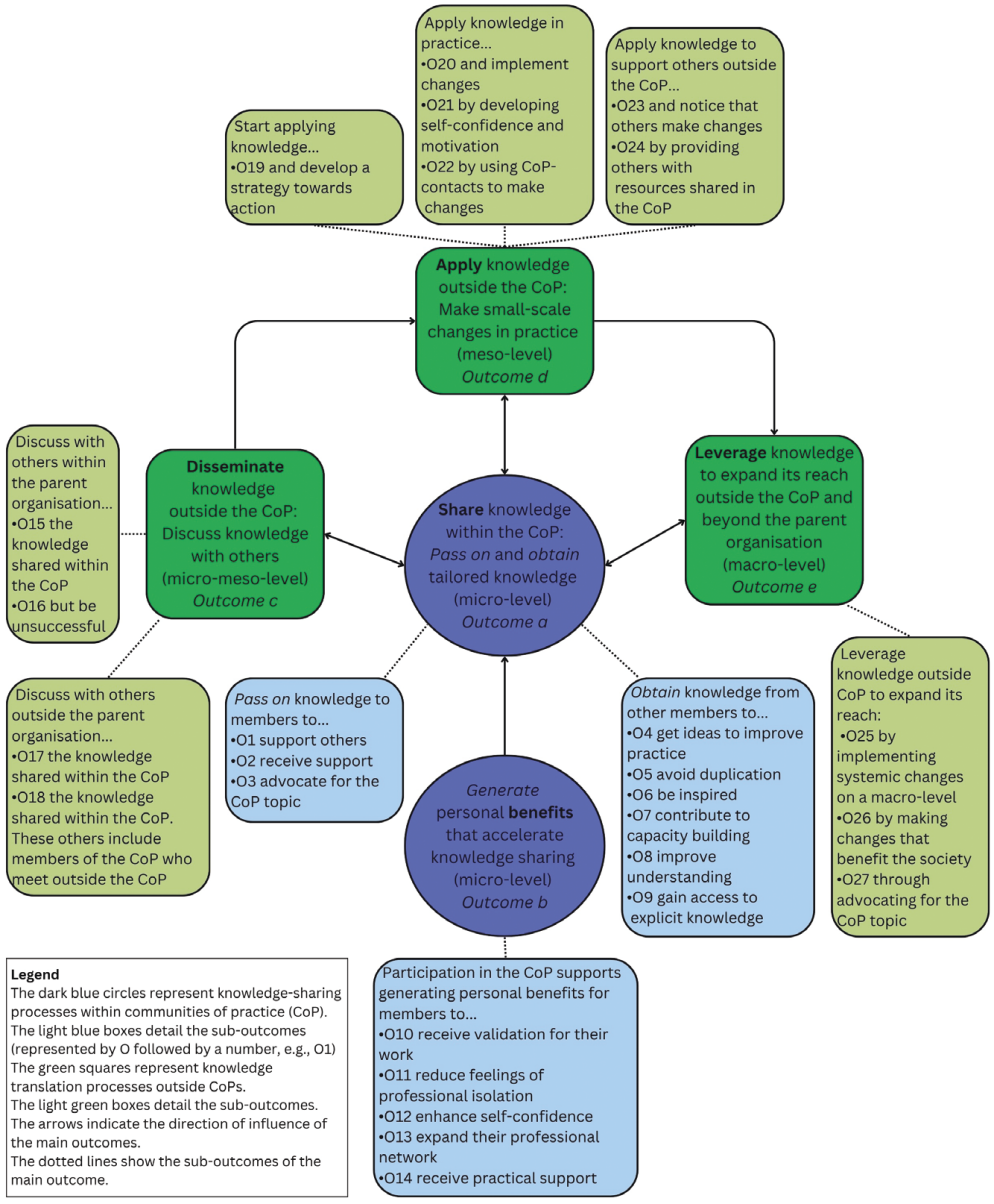
Overview of outcomes reported by interviewees

The five main outcomes were divided into 27 sub-outcomes. Figure 2 presents an overview of all outcomes, including the relationships between the five main outcomes of knowledge sharing *within* and knowledge translation *outside* the communities of practice.

### Micro-level outcomes: Knowledge-sharing within communities of practice

All but one of the 32 interviewees indicated knowledge sharing as a direct outcome of participating in a community of practice. This knowledge sharing was categorized as *Passing on knowledge* to other members (*n* = 18), *Obtaining knowledge* from other members (*n* = 31) (Outcome a) and *Generating personal benefits* that supported knowledge sharing (*n* = 29) (Outcome b). Members stated that this knowledge sharing increased their capacity and created (new) tailored knowledge for them.

#### Passing on knowledge to others (Outcome a)

Eighteen interviewees reported three sub-outcomes related to passing on their knowledge to support other members [Outcome 1 (O1)], to receive support back (O2) or to advocate for the topic, the specific public health issue of their community of practice (O3) (Figure 2). Observations showed that in communities of practice with a relatively large core group (CoP B and C), more members actively shared knowledge by valuing each other’s work, exchanging experiences and providing potential solutions. In CoP A, D and E, with a smaller core group, most members attended infrequently (peripheral members), and discussions typically involved a few core members, while peripheral members mostly listened. All communities of practice included text chatting activities alongside oral discussions. Interactive parallel discussions were observed in CoP C and E. Both engaged a dedicated person to monitor and respond to the chat. Interviewees reported the valuable role of these chat monitors to clarify uncertainties and provide a safe place for those who chose not to speak.

Interviewees passed on their knowledge to support others (O1, *n* = 13) to reciprocate what they obtained from others. Interviewees with ample professional experience expected some benefits from sharing but were also motivated by altruism. Observations confirmed that these experienced members and facilitators actively participated in sessions and were commonly the first to comment, provide answers or ask questions to support less experienced members. Openly discussed failures by experienced members and facilitators led to engaging discussions, with others sharing their challenges.

Interviewees indicated that they passed on their experiences and challenges, intending to receive support or practical assistance for their projects or emotional encouragement and social validation for their work (O2, *n* = 11). Some interviewees suggested that asking questions and sharing experiences was the only way to gain sufficient benefits from the sessions:

*I feel if I don’t ask any questions, I’m not getting anything out of it at all. So, I thought the more I put into it, the more I could get out of it.* (D5)

Initially, the observed practice in sessions was the sharing of successful experiences. However, members became more open over time and started sharing their challenges while actively asking for support. Interviewees confirmed this, and some suggested that online break-out rooms provided options for easier sharing in small groups. In contrast, others reported that break-out room conversations were too shallow due to time spent on introductions.

Interviewees, including members with lived experience and facilitators, passed on their knowledge to advocate for action on public health issues or promote their organization (O3, *n* = 8). Observations showed that members with lived experience raised awareness by sharing personal experiences. These members reported in the interviews feeling optimistic that their efforts may lead to positive changes. This sharing was well-received by other members but not valued more than the experience sharing of other (professional) members. Facilitators reported advocacy activities as a pivotal reason to facilitate the community of practice. They were often observed to provide practical tools and explicit knowledge that were readily applicable in practice.

#### Obtaining knowledge through communities of practice (Outcome a)

Data analysis of 31 interviewees identified six sub-outcomes of obtaining knowledge through participation in a community of practice. Members obtained knowledge that not only provided ideas to improve their practice (O4) but also facilitated avoiding duplication of their work (O5), offered inspiration for (future) improvements of their practice (O6), contributed to the individual members’ capacity building (O7), improved members’ understanding of the public health issue (O8) and facilitated access for members to explicit knowledge (O9) (Figure 1).

Interviewees reported that attending the sessions provided them with ideas to improve their practice now or in the future (O4, *n* = 21). They indicated in interviews that obtaining tacit knowledge (e.g. sharing best practices and challenges) from others with similar experiences was highly beneficial because they could not easily acquire such knowledge in their regular professional environments. It was observed that members voiced their appreciation in sessions for obtaining such knowledge. Some interviewees indicated that obtaining knowledge through communities of practice saved them time, as it helped them avoid work duplication and potentially saved them from making similar mistakes in the future (O5, *n* = 6).

Many interviewees indicated that knowledge shared in communities of practice did not necessarily lead to an intention to make changes but instead served as general inspiration (O6, *n* = 22). A strong interest in the specific topic typically motivated participation in the community of practice. For example:

*It was really interesting to hear about their experiences and to hear about all the different layers that they were working with and sort of dealing with health literacy as an issue. Whether it is in community health or hospitals, allied health or in prisons or even in some of those refugee communities. I thought that was so interesting.* (D4)

Interviewees reported that sharing tacit and explicit knowledge contributed to capacity building (O7, *n* = 19), with tacit knowledge being mentioned more frequently. It also increased interviewees’ understanding of specific public health issues and ongoing projects (O8, *n* = 13). Additionally, interviewees stated that their community of practice facilitated access to otherwise hard-to-obtain explicit knowledge and provided practical support to navigate available tools and resources (O9, *n* = 16).

#### Generating personal benefits (Outcome b)

Engaging in knowledge-sharing processes led to personal benefits for 29 interviewees. The five sub-outcomes in this area were obtaining social validation for their work (O10), reducing feelings of professional isolation (O11), enhancing self-confidence (O12), expanding professional networks (O13) and gaining practical support from others (O14) (Figure 2). While indirectly supporting knowledge translation outcomes, such benefits engendered positive participation experiences that accelerated knowledge sharing and capacity building.

When interviewees shared experiences, they obtained social validation (O10, *n* = 12) through others’ valuing their work. About half of the interviewees stated this was particularly important because working on niche, non-urgent public health topics made them feel professionally isolated. Connecting with like-minded others who shared a similar passion helped reduce these feelings of isolation (O11, *n* = 15).

Participation enhanced interviewees’ confidence in their topic knowledge and ability to take action or improve current and future projects (O12, *n* = 13). This enhanced confidence was supported by opportunities in sessions to connect more experienced members with those less experienced. Active members valued having lurkers—members who listen but do not actively engage—as this was seen as essential support and boosted their confidence, feeling supported by a group of like-minded others.

An expanded professional network was another valued outcome (O13, *n* = 22). Interviewees expected to utilize this network when applying new knowledge in the future. Additionally, some interviewees shared that other members already supported them outside formal sessions, which helped them in their projects (O14, *n* = 12). For example:

*There’s been the development of relationships between the members. So, it is a bit of shared identity going on; it is sort of mutual support, but also just one-to-one relationships as people have helped each other*. (B3)

#### Translation of knowledge to action outside communities of practice

Interview and observational data demonstrated all but one interviewee went beyond knowledge sharing to translate the new or updated knowledge into action in their practice and supporting their work in addressing public health issues by (c) disseminating knowledge to others (*n* = 19), (d) applying it in their practice (*n* = 24) and (e) leveraging the knowledge to expand its reach beyond their own practice (*n* = 11).

### Micro-meso-level outcomes: Disseminate knowledge to others (Outcome c)

Interviewees indicated that they disseminated knowledge outside their community of practice by discussing it within and also outside their parent organization (Figure 2). The interviewees identified these outcomes as easily attainable and feasible to implement in the short term within their organization.

Interviewees reported carefully considering which knowledge was worth disseminating to colleagues and managers within their parent organization (O15, *n* = 10). For example:

*The things that I’m hearing in the community of practice, I was able to bring over some useful information that was presented back to the working group.* (C3)

Some interviewees anticipated that sharing would support them in developing new projects in the future. Two interviewees pointed out they were unsuccessful in sharing knowledge internally (O16, *n* = 2) because their organization was not ready for it, making them feel ahead of their time.

Interviewees also translated the knowledge and disseminated it outside their parent organization. When knowledge dissemination was part of their regular role, interviewees mostly shared tacit knowledge, such as stories and experiences, with external partners (O17, *n* = 4). Interviewees also contacted other members to share explicit knowledge outside the sessions, such as practical tools (O18, *n* = 7). During sessions, some members reported discussing knowledge from previous sessions with external stakeholders. This prompted a response where others asked how the knowledge was perceived and if tips could be shared on effective knowledge dissemination.

### Meso-level outcomes: Apply knowledge to make small-scale changes in practice (Outcome d)

Twenty-four interviewees reported applying knowledge in their practice by developing and implementing changes and supporting others to do the same (Figure 2). Interviewees pointed out that while these outcomes were achievable, their implementation might take some time.

Through their participation in the community of practice, interviewees realized that their organization needed a different approach to address the public health issue, which prompted the development of a strategy for action (O19, *n* = 19). For example, one member used the new knowledge to make their practice more inclusive:

*We go back to the team and discuss then with my team how we can change some of our research practices to ensure we involve everyone or attempt to involve as many people as possible within our population.* (D2)

About half of the interviewees reported using knowledge to make small-scale changes in their practice (O20, *n* = 13). An example was the implementation of training for general practitioners to use trauma-informed care language. Interviewees suggested that participation inspired them and improved their confidence in implementing changes (O21, *n* = 7). For example:

*I felt quite confident and perhaps being a bit inspired to change my direction*. (B5)

Interviewees indicated that the social validation they received for their work from other members encouraged them to make changes (O22, *n* = 7). In the sessions, some members mentioned that they had started applying the new knowledge in their practice. In CoP B, members collaborated to implement activities together. They discussed in the interviews that this collaboration saved time and effort that could be invested in other activities.

Some interviewees reported that, for various reasons, they were not (yet) able to make changes. However, by disseminating knowledge outside their community of practice, they supported others who were in a position to make changes (O23, O24, *n* = 5). In that case, they preferred to disseminate explicit knowledge, which could be immediately implemented in practice.

### Macro-level outcomes: Leverage knowledge to expand reach beyond own practice (Outcome e)

About a third of the interviewees reported outcomes that extended beyond changing their practice, leveraging knowledge to expand its reach (Figure 2). Interviewees identified these outcomes as long-term outcomes that needed time to be implemented and were typically still in the early stages at the time of the interview.

The interviewees leveraged the shared knowledge from the sessions to initiate changes that ultimately aimed to improve health outcomes. Some interviewees indicated they utilized the shared knowledge to scale up changes (O25, *n* = 3). For example, one interviewee developed programmes that enhanced inclusive behaviour in doctors, aiming to create better clinical outcomes for people with disabilities. Other interviewees reported that the shared knowledge supported their projects and that the outcome of these projects reached beyond their own organizations (O26, *n* = 4). For example:

*It is not just about doing an amazing project that does impact people on the ground (…). I spoke to my consumer group, and we want to do this because of this issue. And I think it kind of reiterates why we are doing what we are doing. And it helps remind you that these are real issues that have on-the-ground impacts.* (B4)

Five interviewees mentioned that the shared knowledge helped them to advocate for the specific public health issue (O27, *n* = 5). For example, an interviewee leveraged the knowledge to successfully advocate for implementing trauma-informed care practice in a physiotherapy curriculum.

### Expected and actual outcomes of communities of practice

During the interviews, two-thirds of the interviewees (*n* = 22), who had also participated in the needs assessment, reviewed their initial expectations. All except one reported that participation in the community of practice met their expectations. The other 10 interviewees did not complete the needs assessment. Although most of them still reviewed their expectations, their actual outcomes could not be compared with the initial expectations. Table 3 provides a detailed comparison of the needs assessment expectations of 22 interviewees with the outcomes they reported in the interviews. Outcome b, generating personal benefits, was not identified as an expectation by participants during the needs assessment and is therefore not included in Table 3.

**Table 3:** Comparison of initial expectations versus actual outcomes reported in the interviews

	Outcomes reported in interviews				
**Expected outcomes reported in needs assessments**	**Share knowledge within the CoP** *Outcome a*	**Disseminate knowledge outside the CoP** *Outcome c*	**Apply knowledge outside the CoP** *Outcome d*	**Leverage knowledge outside the CoP** *Outcome e*	**Total**
Total interviews	32	19	24	11	32
Number of interviewees that completed the needs assessment	22	13	15	7	22
**Short-term expectations reported in needs assessment**					
Developing increased knowledge	17	9	12	6	17
Start action on public health issue	5	3	2	0	5
Build connections between members	8	7	7	3	8
Improving health outcomes	0	0	0	0	0
Receive support from others	2	1	2	0	2
**Long-term expectations reported in needs assessment**					
Having increased knowledge	6	4	4	2	6
Taking action on public health issue	10	4	7	1	10
Having connections with members	12	7	10	4	12
Improving health outcomes	9	5	6	2	9
Receiving support from others	2	2	2	1	2

Interviewees who expected to increase their knowledge in the short term through participation reported achieving this and also translating it beyond the community of practice. Those expecting short- or long-term outcomes of making connections typically reported knowledge translation outcomes. However, only half of those who expected to build or have connections reported actually connecting with others. Those who expected action outcomes in the long-term outcomes also frequently reported these knowledge translation (action) results. Those expecting improved health outcomes for their patients in the long term rarely reported this as an actual outcome.

Most interviewees stated that their community of practice met their expectations of knowledge sharing, learning and connecting with others. Specifically, members’ expectations of action (knowledge translation) strongly align with actual reported outcomes. For example:

*That [short-term collaboration and sharing, and long-term developing networks and engagements] is exactly what came out of it. And collaboration…. I don’t even know what I meant by collaboration then, but that actually ended up being a massive outcome of this work.* (B4)

When low expectations were reported in the needs assessment, interviewees typically stated that participation exceeded their expectations. For example, some interviewees initially preferred face-to-face sessions, but the geographical dispersion of members and COVID-19 pandemic restrictions forced the communities of practice to run online. Interviewees indicated more positive outcomes than expected of online sessions due to having easier access to experts and allowing a more diverse group to participate. They suggested that more diversity led to more tacit knowledge sharing and more options for knowledge translation. Some interviewees found their community of practice useful yet different than expected. External circumstances also affected some interviewees’ ability to achieve their expected outcomes. One interviewee suggested that the community of practice was not meeting their expectations, but they kept participating and remained hopeful for the future.

In the co-design process, the needs assessment urged interviewees to identify their expectations beforehand. Some interviewees stated that identifying helped them to make their objectives explicit, contrary to the usual process where they joined without making their expectations explicit. Interviewees from all five communities of practice appreciated that facilitators were attentive to their needs and that they valued that they could present their needs at any time. However, besides the needs assessment, most members did not take up this opportunity. Open calls for input from facilitators typically resulted in minimal responses. Yet direct individual invitations to contribute to sessions often received a positive response.

All facilitators indicated that they benefitted from using the co-design method, especially the needs assessment, which supported facilitators in meeting members’ needs, even when they had to prioritize these needs over their own. For example:

*I think if they [members] had come and said: ‘All we want to do is talk to each other’, we would have had a completely different approach. We would then just have a hands-off approach. […] Because sharing and gaining knowledge was one of the key things in the needs assessment, we felt obligated to provide some sort of education or information […]. Absolutely. I think it influenced us.*

Observations showed facilitators generally followed the needs assessment results, particularly at the start. While one facilitator claimed they could not remember using the needs assessment, the research team observed, and interviewees confirmed that the facilitator acted along the lines of the needs assessment results.

## DISCUSSION

This study aimed to describe the outcomes of co-designed communities of practice that support members in addressing public health issues. The study sought to provide insights for future communities of practice while addressing the need for more robust evaluations of communities of practice that focus on public health issues (e.g. Barbour *et al.*, 2018; James-McAlpine *et al.*, 2023). Members reported outcomes that supported their public health practice in health literacy, mental health literacy and trauma-informed care. Specifically, members shared knowledge within communities of practice and generated personal benefits. Members disseminated, applied and leveraged that knowledge outside communities of practice, resulting in outcomes within and beyond their organizations. These findings are important because they provide comprehensive and detailed insights into the outcomes of communities of practice addressing public health issues. The insights fill a knowledge gap and will support the development and impact of future communities of practice.

This study identified five outcomes for community of practice members that support addressing public health issues. The first two micro-level outcomes occur within communities of practice where members share knowledge and generate personal benefits. To share knowledge, reciprocity, as described in social exchange theories (Cropanzano and Mitchell, 2005), was essential for all members to justify their participation. This extends insights from previous studies (e.g. Ivcovici *et al.*, 2022), which suggested that reciprocity was particularly crucial for members with significant professional experience to avoid disengagement. Our findings show that most members, including facilitators, members with substantial professional experience and members with lived experience, shared their knowledge to receive support or gain benefits later. Supporting others was seen as a secondary, less relevant outcome. Future facilitators may consider strongly supporting this desire for reciprocity as a condition for effective communities of practice.

Three outcomes were identified where members indicated that they translated the shared knowledge, attesting that community of practice members can go beyond knowledge sharing and move towards action. Several studies suggested micro- and meso-level outcomes where members disseminated knowledge to their parent organizations and changed their practice (e.g. Chandler and Fry, 2009; Jennings Mabery *et al.*, 2013; Poole and Bopp, 2015). Our study supports this and extends prior findings by showing that members of co-designed communities of practice also disseminated the knowledge beyond their parent organization, widening the impact. It also expands knowledge by providing new insights where the sharing of tacit knowledge supported building confidence and motivation, which positively contributed to implementing practice changes. While some previous studies stated improving health outcomes as a potential macro-level outcome, the existing empirical evidence was not strong (e.g. Chandler and Fry, 2009; Jennings Mabery *et al.*, 2013; Poole and Bopp, 2015). In our study, a third of the interviewees reported outcomes beyond changing practice, potentially improving health outcomes for some. Interviewees recognized that these outcomes might be premature, and systemic changes could take years. They suggested that communities of practice provided new knowledge and access to support, which could create more impact in the future. This implies that communities of practice may not be a short-term quick solution for improving health outcomes, but they may prove effective as a gradual, longer-term solution.

Our study evaluated if embracing co-design rather than top–down designed communities of practice fulfilled members’ needs and mitigated the risk of them not fulfilling their potential—a frequently observed weakness of communities of practice (Gabbay *et al.*, 2003; Wehrens *et al.*, 2012; Bindels *et al*., 2014). Comparing the initial needs assessment results (Elbrink *et al.,* 2023) with the actual outcomes showed, for the first time, that co-designed communities of practice meet members’ needs. The co-design methods also appeared to optimize members’ efforts beyond simple knowledge sharing to generate knowledge translation. Initial evidence indicated that some members started to improve health outcomes by making changes in and beyond their practice based on shared knowledge. The co-design method was reviewed as useful by all facilitators, not considered burdensome by members, and also identified as beneficial by some interviewees. This suggests that co-design is a useful way for policymakers, professionals and researchers to implement effective communities of practice.

## STRENGTHS AND LIMITATIONS

Even though the setting of communities of practice and the researchers’ influence differed during the study, the findings of our research were consistent within and across the five communities of practice. This demonstrates strong validity through prolonged, longitudinal involvement, data triangulation (interviews and observations) and respondent validation through subsequent interviews. Our consistent and transparent methods, along with detailed findings, enhanced the study’s rigour and robustness, leading to credible results. Reliability was ensured through constant data comparison and peer triangulation. Transferable generalisability was supported by context descriptions of the communities of practice and purposeful sampling, allowing future researchers to compare their contexts with ours. While different contexts of communities of practice in the future may shed more light on our findings, the potential generalizability of our findings, the knowledge translation outcomes, have the potential to extend to other contexts.

We recognize that the findings may have been affected by the low representation of some types of members, including those who have difficulties related to low digital skills, access, language and time differences. The potential influence of the researchers in the co-design process was mitigated, as much as possible, by varying involvement across the communities and by ensuring transparency and consistency in our methods. Future research can be further deepened by proactively including different types of members and communities of practice in other contexts and by minimizing researcher impact during the co-design process. A manual has been drafted to enable implementations of the co-design method (available from the authors upon request).

## CONCLUSION

This study sought to provide insights for developing future communities of practice that focus on public health issues and respond to calls for more robust evaluations. The study provided strong evidence that knowledge sharing within communities of practice led to members translating the knowledge outside communities of practice to generate effective responses to public health issues. The novel co-design method was positively received and helped to ensure communities of practice align with members’ needs. The evidence generated in this study includes guidance that will assist policymakers, professionals and researchers in implementing communities of practice that meet members’ expectations and go beyond knowledge sharing and translation to improve public health.
